# Intimate Partner Violence, Legal Systems and Barriers for African American Women

**DOI:** 10.1177/08862605221090561

**Published:** 2022-04-20

**Authors:** Ellen R. Gutowski, Stephanie Freitag, Shujing Zhang, Martie P. Thompson, Nadine J. Kaslow

**Affiliations:** 1 7938 University of Toronto, ON, Canada; 2 12239 Emory School of Medicine, Atlanta, GA, USA; 3 1801 Appalachian State University, Boone, NC, USA

**Keywords:** African American women, intimate partner violence, legal systems, barriers to services, hopelessness

## Abstract

Although many African American IPV survivors need services, they often do not access care. Hopelessness may partially explain low rates in help-seeking for this population and serve as a significant barrier to care for African American IPV survivors particularly those who have had prior legal system involvement. In a sample of 185 African American women, we first examined whether hopelessness mediated the relation between IPV and barriers to services. If such a mediation effect was found, we then would explore whether legal system involvement moderated the mediated effect of hopelessness on the relation between IPV and barriers to services. As anticipated, hopelessness partially served to explain (i.e., mediated) the relation between IPV and barriers to services. Further, this mediated effect was moderated by legal system involvement such that when legal system involvement was included as a moderator, hopelessness mediated the association between IPV and barriers to services *only* for those survivors who had been involved with the legal system. These results underscore the critical role of hopelessness as a barrier to accessing services for African American IPV survivors, especially those with prior involvement with the legal system. Recommendations are offered that underscore the importance of interventions that empower African American women who have survived violence instead of penalizing them.

Intimate partner violence (IPV) is a widespread global health problem. Intimate partner violence has multifaceted and wide-ranging effects on survivors, since being psychologically, sexually, and/or physically abused by an intimate partner often creates challenges in multiple domains of their lives (Dillon et al., 2013). While IPV is a problem for individuals from all sociodemographic backgrounds, some groups are disproportionately affected. Four in ten Black women in the United States (US) experience IPV throughout their lifetime (Black et al., 2011). As a consequence of exposure to multiple forms of oppression, African American women in particular, especially those who come from low socioeconomic status backgrounds, experience high rates of severe and recurrent IPV (Lacey et al., 2016). As a result, they are at elevated risk for mental health problems including post-traumatic stress, depression, substance use, and suicidality (Mugoya et al., 2020; Sabri et al., 2013; Watson-Singleton et al., 2020). Furthermore, due to the intersections of racism and IPV, African American IPV survivors often face poverty and associated difficulties (e.g., homelessness, unemployment), which compound the negative physical and mental health effects of interpersonal violence (Gillum, 2019; Mugoya et al., 2020).

Given the pernicious effects of IPV, survivors often have a substantial need for services. Social services (e.g., housing, food banks, disability, and employment) may be crucial for ensuring safety and stability for those facing the economic consequences of IPV (Clark et al., 2019; Dichter & Rhodes, 2011). Comprehensive healthcare is essential for addressing the physical and mental health sequelae of IPV (Dichter & Rhodes, 2011). Unfortunately, however, IPV survivors often are unable or hesitant to seek help (Robinson et al., 2020) and this is commonly the case for African American women (Waller et al., 2021). In addition, African American IPV survivors report more interest in obtaining economic resources, long term housing support, and healthcare, than services that are traditionally offered to them (i.e., law enforcement, temporary housing, and counseling), suggesting a mismatch between typically available services and the needs of these survivors (Dichter & Rhodes, 2011; Gillum, 2008). Moreover, African American survivors are less likely than women from other ethnoracial groups to disclose their victimization experiences to professionals (Postmus, 2015), potentially because many service providers lack the training and experience to offer culturally responsive services (Gillum, 2008; Waller et al., 2021). For example, providers often do not understand how race shapes experiences of IPV, which leaves many African American women feeling othered (Gillum, 2008). In a related vein, African American women report concerns about seeking help from police due to feelings of mistrust given the long history of racist law enforcement practices within the US (Gillum, 2008).

In the context of these systemic constraints, barriers to help-seeking commonly experienced by IPV survivors may be explained partially by hopelessness (Nathanson et al., 2012). There is some empirical evidence that supports this contention (Han et al., 2018). Hopelessness theory defines hopelessness as a system of negative expectations about oneself and one’s future (Abramson et al., 1989; Beck et al., 1974). Those experiencing hopelessness anticipate that desirable outcomes are unlikely, expect that one will be met with undesirable outcomes, and harbor a sense that they are helpless to change the likelihood of these outcomes (Abramson et al., 1989). Hopelessness theory postulates hopelessness as a core feature of depression that is brought on by the stress of negative life events (Abramson et al., 1989). Consistent with this, empirical findings support hopelessness as a risk factor for mental health symptoms and disorders, such as depression and post-traumatic stress, among interpersonal violence survivors (Scher & Resick, 2005). Furthermore, within the African American community, hopelessness is a sequelae of race-related stress (Odafe et al., 2017). Among African American women who have survived IPV, depression is highly prevalent (Mugoya et al., 2020) and hopelessness is a serious yet under-explored problem. Hopelessness is associated with increased suicidal behavior and mediates the IPV-suicide attempt link for these women (Thompson et al., 2002). Hopelessness may be key to the low rates of help-seeking among African American survivors of IPV; it may impede their ability to seek needed services as they do not believe such services will be accessible or helpful (Wilson et al., 2007). For African American survivors, especially those who are low-income, being unable to obtain needed health, mental health, and social services may increase their sense of hopelessness about coping with the psychosocial and economic challenges that result from IPV. Moreover, they may develop the view that seeking help from law enforcement personnel in response to IPV victimization is futile, given frequent experiences and reports in their communities and in the popular press of not being taken seriously or being met with police violence (Decker et al., 2020).

One group of African American women IPV survivors for whom the association among IPV, hopelessness, and barriers to help-seeking may be pronounced are those with a history of legal system involvement. African American IPV survivors have a complex legacy of involvement with the legal system (Richie, 2012). Inequities within the US legal system result in African American survivors having an increased likelihood of criminal charges and incarceration (Richie, 2012). Indeed, some African American IPV survivors are racially profiled and met with criminal charges in the context of seeking help from law enforcement when they have never committed a crime or their “crime” was an attempt at survival or a response to the abuse (Kim, 2018; Richie, 2012). Unfortunately, in addition to racially biased determinations, courts often fail to understand the psychosocial factors that surround survivors’ actions, resulting in unfair convictions and time in prison for many African American IPV survivors (Kim, 2018).

African American survivors with a history of legal system involvement may face unique barriers to obtaining adequate services (Richie & Eife, 2020). Processes that criminalize rather than protect IPV survivors with a history of incarceration often reduce their access to necessary resources, a pattern found to disproportionately impact African American women (Richie & Eife, 2020). In addition, when they seek help from law enforcement, these women often are mistreated and devalued, which further increases their hesitancy to seek professional assistance. Unfortunately, no research could be located that examines hopelessness in African American IPV survivors with a history of involvement in the criminal legal system.

## Current Study

Intimate partner violence is associated with increased barriers to services and can foster hopelessness. Hopelessness in turn may magnify barriers to services experienced by survivors. These associations may be more pronounced among IPV survivors with a history of legal system involvement than their counterparts without such a history. Hopelessness theory postulates that hopelessness is brought on by negative life events and functions as a core feature of a subtype of depression (Abramson et al., 1989). Yet, little research has examined if hopelessness mediates the link between IPV and barriers to services for African American women in general or plays a unique role in explaining this association among legally involved IPV survivors. To fill these gaps, the present study examined whether hopelessness mediates the relation between IPV and barriers to services, and tested a moderated-mediation model to explore whether legal system involvement moderates the mediated effect of hopelessness on the relation between IPV and barriers to services. We hypothesized that hopelessness would mediate the IPV—barriers to services link for the full sample. We predicted that legal system involvement would moderate the mediated effect of hopelessness on the relation between IPV and barriers to services, such that hopelessness would have a greater influence on the IPV-barriers to services link for those who were legally involved compared with those who were not. If the findings are consistent with these hypotheses, they will have important clinical and system level implications.

## Methods

### Participants

Participants included 185 African American women ages 18–59 (M = 36) recruited from a public safety net and academic health system-affiliated hospital serving a low-income, urban population that is largely African American. We collected these data as part of a larger study on IPV and suicidal behavior among low-income African American women (Kaslow et al., 2010). We received referrals from hospital staff and recruited participants from throughout the health system. We invited to participate those who met criteria (self-identified as African American/Black, experienced IPV and made a suicide attempt in the past 12 months).

Among these 185 participants, 114 (61.62%) reported prior involvement in the legal system and 71 (38.38%) denied such involvement. Of those who endorsed prior legal system involvement, 110 (96.49%) previously were incarcerated most often for aggravated assault, battery, use or possession of controlled substances, prostitution, disorderly conduct, trespassing, endangering children, and theft. The sample was low-socioeconomic status as reflected in the following. Participant education levels ranged from less than 12th grade to college degree, with more than half (127; 68.6%) earning a high school diploma or less. Their household incomes were largely below the poverty level; 104 (56.2%) reported a household income of $249 or less per month, 19 (10.3%) earned $250–499, 46 (24.9%) earned $500-$999, 10 (5.4%) earned $1000–1999, and three (1.6%) earned more than $2000. In addition, the participants were primarily unemployed (*n* = 159; (85.9%)) and uninsured (*n* = 116 (62.7%)) and over half (*n* = 96 (51.9%)) considered themselves to be homeless.

### Measures

#### Demographics.

Participants completed a short questionnaire that included questions about demographic information including age, education level, income, and housing. This questionnaire also included the questions: “Have you ever been involved in the legal system?” “Have you ever been in jail or prison?” And, if the participant answered yes to the previous question, they were invited to respond to the open-ended question, “What was the charge?”

#### Intimate Partner Violence.

To examine physical, sexual, and psychological abuse enacted by an intimate partner, participants completed the 30-item Index of Spouse Abuse (ISA) (Hudson & McIntosh, 1981). They rated on a 5-point scale ranging from “never” to “very frequently” the degree to which they experienced the various abusive acts in a present relationship with a partner. Example items include, “My partner belittles me” and “My partner makes me perform sex acts I do not enjoy or like.” For the present study, a large number (*n =* 58) of participants had no children, therefore, the one item asking about being forced to stay home with the kids was not included in the scoring for this scale. The ISA has evidenced excellent internal consistency reliability in prior research with low-income, African American samples (α = .91) (Bradley et al., 2005). The 29 items included in this scale for the present study had excellent internal consistency reliability (α = .97).

#### Hopelessness.

To provide information about their feelings of hopelessness the participants completed the 20-item Beck Hopelessness Scale (Beck et al., 1974), a self-report measure on which they indicated whether statements reflect their attitudes in the past week. Participants responded “true” or “false” to a series of statements. Their responses were summed to create a continuous variable. Sample items include, “Things just won’t work out the way I want them to” and “I might as well give up because there’s nothing I can do about making things better for myself.” This scale has shown excellent internal consistency reliability in prior studies with samples of low-income, African American women (α = .94) (Kaslow et al., 2006). For this study, the scale had excellent internal consistency reliability (α = .93).

#### Barriers to Services.

The Barriers to Accessing Services Scale (BASS) is an 18-item self-report measure that captures various barriers participants face to accessing services. Given the lack of available measures to assess this construct in this population when the study was launched, this measure was adapted from a scale assessing barriers to care in HIV/AIDS populations (Heckman et al., 1998). Modifications were made based on findings from studies assessing barriers to care (Fox et al., 2001; Yeh et al., 2003), including among abused women (Baker et al., 2003). Participants indicated on a 4-point scale ranging from “No problem” to “Major problem” the extent to which each item made it difficult for them to obtain care, services, or opportunities. Higher scores indicate more barriers to receiving services in the community. Sample items include, “Concern with being treated unkindly or unfairly” and “Lack of personal financial resources.” Once again, one item asking about childcare was excluded from the scoring of this measure given the sizeable number of participants who had no children. For this study, the 17 items included in this scale had good internal consistency (α = .89).

### Data Analysis

#### Missing data.

All participants (*n =* 185) who completed measures of interest in the present study were included in the analyses. Among these participants, missing data were minimal: 17 women had missing data and 0.15% of the overall data points were missing. We used Little’s Missing Completely at Random (MCAR) test to ascertain if data were missing completely at random (Little & Rubin, 2019). Results were non-significant (*X*^*2*^ (843) = 907.001, *p* = .062), suggesting that data were missing completely at random. We used multiple imputation to address the missing data; multiple imputation is advantageous for clinical research with a mix of categorical and continuous variables as it employs a flexible assumption about the nature of missingness that reduces the potential for bias in the results (Enders, 2017).

#### Power Analysis.

To determine whether our sample was of adequate size to conduct a moderated-mediation analysis with one mediator and one moderator, we performed an a priori power analysis with a specified power of .95, alpha level of .05, and an effect size of .15 (medium effect size for multiple regression analysis) (Wen & Fan, 2015). Our power analysis yielded a recommended sample of at least 119 participants to achieve power of .95, indicating that our sample size was acceptable.

#### Analytic Plan.

**Figure 1.**
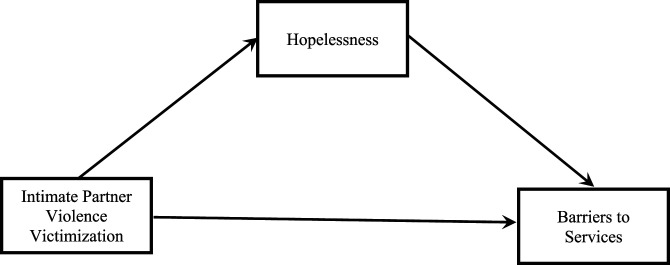
Mediation model with hopelessness as the mediator.

**Figure 2.**
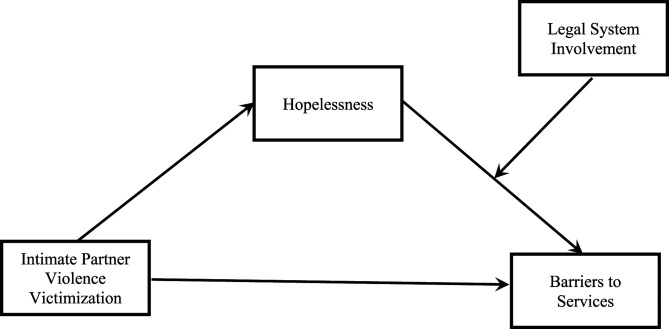
Moderated-mediation model with hopelessness as the mediator and legal system involvement as the moderator.

We used SPSS version 27.0 with Hayes’ PROCESS (Hayes, 2018) extension that allows for a regression-based approach to moderated-mediation analysis with bootstrapping. We ran two models. First, we ran a mediation analysis with the entire sample with IPV as the predictor, barriers to services as the outcome, and hopelessness as the mediator. Next, we ran a moderated-mediation model with hopelessness as the mediator and legal involvement as the moderator between the mediator (hopelessness) and the outcome (barriers to services). See Figures 1 and 2 for visual depictions of each model. Hayes’ extension was a favorable approach for these analyses, as bootstrapping assesses for both indirect and direct effects of variables while maximizing power (Hayes, 2018). To test each model, we conducted bootstrapping with 10,000 replications, as increasing the number of replications increases the stability of estimates (Hayes, 2015).

## Results

### Preliminary Analyses

First, data were screened and assumptions were tested. Data met criteria for normality (skewness <3; kurtosis <10). Continuous variables of interest correlated with one another in the expected directions. Table 1 reports correlations for all continuous variables of interest.

**Table 1.** Correlation Analyses and Descriptive Statistics.

	*n*	*M*	*SD*	Intimate Partner Violence	Hopelessness	Barriers to Services
Intimate partner violence	185	87.11	31.39	––		
Hopelessness	185	8.04	6.03	.26**	––	
Barriers to services	185	39.22	11.61	.26**	.30**	––

*p < .05; ** *p* < .001.

### Mediation Analysis

Table 2 presents results for the mediation analysis with IPV as the independent variable, hopelessness as the mediator, and barriers to services as the outcome variable. As anticipated, IPV (95% CI [.0222, .0764]) had a direct effect on hopelessness (*R*^2^ = .07; F (1,183) = 12.92; *p* = .0004). Further, IPV (95% CI [.0179, .1228]) and hopelessness (95% CI [.2127, .7583]) each had direct effects on barriers to services (*R*^2^ = .12; F (2,182) = 12.91, *p* < .0001). Both models produced an intermediate effect size (Cohen’s d = .53 and .75, respectively) (Cohen, 1988) that fell within the zone of desired effects (Hattie, 2009). Finally, IPV had an indirect effect on barriers to services via hopelessness (95% CI [.0078, .0456]). Thus, consistent with our hypothesis, hopelessness partially mediated the relation between IPV and barriers to services.

**Table 2.** Mediation Analysis: Intimate Partner Violence (IPV), Hopelessness and Barriers to Services.

Variable	Coefficient	SE	*p*	t	LLCI	ULCI
IPV	.0704	.0266	.0088	2.6488	.0179	.1228
Hopelessness	.4855	.1383	.0006	3.5116	.2127	.7583
Indirect effects of IPV on barriers to services						
	Effect	SE			LLCI	ULCI
Hopelessness	.0239	.0098			.0078	.0456

*Note. R*^2^=.12; F (2,182) = 12.91; *p* < .0001.

### Moderated-Mediation Analysis

Table 3 presents the results for the simple moderation model with IPV as the independent variable and hopelessness as the outcome variable. When examining if legal system involvement moderated the IPV exposure—hopelessness link, we found the interaction term to be nonsignificant. However, when looking at different levels of the moderator, for those who were previously legally involved, the relation between IPV exposure and hopelessness was supported (95% CI [.0357, .1042]), whereas for those with no legal system involvement no such relation emerged (95% CI [-.0278, .0592]). The overall model was significant (*R*^2^=.09; F (3,181) =5.81; *p* = .0008) and produced an intermediate effect size (Cohen’s d = .62) (Cohen, 1988) that fell within the zone of desired effects (Hattie, 2009). For the full moderated-mediation model, the direct effect of IPV exposure on barriers to services was significant (95% CI [.0179, .1228]). Bootstrap confidence intervals confirmed the indirect effect of hopelessness (effect = .0340; 95% CI [.0129, .0623]) on this association for those who were legally involved. However, for those who had no prior legal involvement, there was no such indirect effect (effect = .0076; 95% CI [-.0114, .0310). Table 4 overviews the conditional indirect effects. The model tested presented a significant index of moderated mediation (index = .03, 95% CI [.0004, .0587]). Further, the overall model was significant (*R*^2^=.12; F (2,182) =12.91; *p* < .0001). The effect size for the model was intermediate (Cohen’s d = .75) (Cohen, 1988) and fell within the zone of desired effects (Hattie, 2009). Thus, for legally involved but not for non-legally involved survivors, experiencing more hopelessness was associated with endorsing more barriers to services when they had more frequent IPV exposure.

**Table 3.** Moderation Analysis: Intimate Partner Violence (IPV), Legal System Involvement, and Hopelessness.

Variable	Coefficient	SE	*p*	t	LLCI	ULCI
IPV	.0157	.0220	.4782	.6909	−.0278	.0592
Legal system involvement	−4.0410	2.5917	.1207	−1.5770	−9.1549	1.0729
IPV X legal system involvement	.0543	.0281	.0545	1.9547	−.0011	.1097
Conditional effects of the focal predictor (IPV) at values of the moderator (hopelessness)						
	Effect	SE	*p*	t	LLCI	ULCI
Not legally involved	.0157	.0220	.4782	.7107	−.0278	.0592
Legally involved	.0700	.0174	.0001	4.0292	.0357	.1042

*Note. R*^2^=.09; F (3,181) =5.81; *p* = .0008.

**Table 4.** Moderated-Mediation Analysis: Intimate Partner Violence (IPV), Legal System Involvement, Hopelessness, and Barriers to Services.

Variable	Coefficient	SE	*p*	t	LLCI	ULCI
IPV	.0704	.0266	.0088	2.6488	.0179	.1228
Hopelessness	.4855	.1383	.0006	3.5161	.2127	.7583
Conditional effects of the focal predictor (IPV) at values of the outcome variable (barriers to services)						
	Effect	SE			LLCI	ULCI
Not legally involved	.0076	.0105			−.0114	.0310
Legally involved	.0340	.0129			.0120	.0623

*Note. R*^2^=.12; F (2,182) =12.91; *p* < .0001.

## Discussion

The results of this investigation advance our understanding of barriers to services among low-income, African American women who are survivors of IPV. This is the first study to examine hopelessness as a mediator of the IPV-barriers to services link in this population and to ascertain if legal system involvement moderates this link. The findings offer compelling support for the key role that hopelessness plays in the process through which IPV is associated with increased barriers to services among this population. They also highlight the effect of a history of legal system involvement on the association between IPV and barriers to services. Indeed, hopelessness was only a mediator of this link for those survivors who have been involved in the legal system.

Consistent with evidence that hopelessness is a common response to IPV (Pathak et al., 2019) and often associated with increased psychological distress in abused women (Kisa et al., 2019), including those who are African American (Pickover et al., 2018), this study offers the first empirical support of hopelessness as a mediator in the IPV exposure—barriers to services link. African American women who have survived IPV too often must cope with the effects of violence on their lives without relevant and needed services and supports. When they do access care, these women routinely are dismissed, devalued and mistreated by providers in the legal, shelter, and healthcare systems—the very systems tasked with assisting them at times of vulnerability (Waller et al., 2021). Indeed, African American survivors report receiving subpar services relative to European American survivors and facing racial microaggressions when seeking care in the aftermath of violence (Gerassi, 2020; Waller et al., 2021). In the context of extreme barriers to services, it is no wonder that African American survivors report high levels of hopelessness, as hopelessness entails an expectation of negative results and is associated with experiences with racism (Mitchell et al., 2020).

Supporting our understanding that negative systems-level interactions shape hopelessness, data from this investigation reveal that the mediating effect of hopelessness in the IPV-barriers to services link was moderated by legal system involvement. Specifically, hopelessness partially explained the IPV-barriers to services association, but only among those survivors with prior legal system involvement. As the legal system is notorious for mistreating African American women, it is not surprising that harboring feelings of hopelessness magnifies barriers to care for those who have been legally involved (Potter, 2009; 2010; Ritchie, 2017). Overall, these results extend prior literature, illuminating the critical role of prior legal system involvement in the mental health of African American women who have survived interpersonal violence. Formerly incarcerated African American IPV survivors may have unique mental health concerns and service needs that differ from African American IPV survivors who have had no prior involvement with the law. For example, they may have been subjected to greater exposure to distressing experiences beyond incarceration including traumatic events while in jail, racial trauma, and/or institutional betrayal (i.e., experience of harm perpetrated by the institutions tasked with offering safety and healing) (Richie & Eife, 2020; Smith & Freyd, 2014). As a result, consistent with hopelessness theory, hopelessness depression (Abramson et al., 1989) may be especially prevalent among legally-involved African American IPV-exposed women. Unfortunately, the implications of these findings are that such experiences may adversely affect African American survivors’ ability to approach other systems of care. Thus, just as incarceration has been framed as a social determinant of health for African American men in the US (Nowotny & Kuptsevych-Timmer, 2018), the same may be argued to be the case for African American women.

There are several potential explanations for the unexpected finding that hopelessness did not mediate the relation between IPV and barriers to services for women with no prior legal system involvement. First, compared to their peers with a history of legal system involvement, women without such a history may have higher levels of protective factors that buffer them against feelings of hopelessness, such as personal and interpersonal resilience and/or social support (Collazzoni et al., 2020; Odafe et al., 2017). Second, this subpopulation may experience systemic constraints (e.g., lack of transportation) as posing greater barriers to services than psychological constraints (e.g., hopelessness). Third, the absence of traumatic experiences of conviction and incarceration may have produced lower levels of hopelessness than seen in survivors with a history of legal system involvement with marked exposure to traumatic stress and institutional betrayal.

The fact that mediation only emerged for IPV exposed women with a history of legal involvement echoes calls to attend to the unique impact of legal system involvement on this specific group of African American women (Kim, 2018; Richie & Eife, 2020; Ritchie, 2017). These findings corroborate extant scholarship suggesting that African American IPV survivors in search of safety may instead encounter harmful systemic responses and that they may be doubly harmed when met with violence from law enforcement personnel or wrongful incarceration in the context of help-seeking (Richie & Eife, 2020; Ritchie, 2017; Waller et al., 2021). Yet, despite the potential for devastating psychological consequences, the dual traumas of IPV and incarceration that affect large numbers of African American women have been vastly under-acknowledged in psychological research and practice. Thus, a major contribution of the current research is that for African American women criminalized amid coping with the traumatic stress of their partners’ abuse, hopelessness exacerbates their barriers to services.

While this study makes a significant contribution to the current literature, several limitations are important to consider. First, study recruitment depended on referrals and follow-through and there may be systematic differences between women who were and were not referred, as well as between those who followed through and participated in the research and those who did not. Such factors are critical to attend to given that African American survivors are less likely to disclose victimization experiences to providers than are those from other ethnic groups (Postmus, 2015). Second, because the data are cross-sectional, we cannot attribute causation or establish temporal precedence for the relation between IPV and barriers to services. However, we can interpret putative relations with caution because mediation is a useful tool for discovering possible causal relations without inferring causality (Agler & De Boeck, 2017). Third, several measurement issues reflect potential methodological weaknesses. We developed the measure to assess barriers to services for the present study. Thus, it was not previously validated. In addition, this measure assessed only participants’ perceptions about barriers to services not actual service use. Further, our measure of legal system involvement did not capture key details of participants’ interactions with the legal system (e.g., length of legal involvement, type of legal involvement, parole restrictions, violence acceptance), many of which have been shown to be important factors in post-incarceration transitions for African American men (Oliver & Hairston, 2008). Heterogeneity in how legal system involvement was defined may introduce confounds that were unaccounted for in this study. Fourth, data on factors that likely play a role in barriers to services (e.g., racism, discrimination, access to resources) and those variables that are potential correlates of hopelessness (e.g., depression, PTSD, suicidality) were not accounted for in this study. It is likely that hopelessness partially explained the IPV-barriers to services relation in combination with system-level factors, such as poverty and racism. Moreover, in the present study, we did not examine psychological and emotional experiences, such as fear and mistrust that may be key to understanding African American women’s barriers to accessing care.

Despite the aforementioned methodological weaknesses, the findings have implications for research, practice, and policy/advocacy. Future research should explore the unique mental health concerns and service needs of African American IPV survivors with legal system involvement using diverse sampling strategies and methodologies, as well as robust and validated measures of variables of interest. Such research would be strengthened if both psychological and systems-level factors were examined. With regard to practice, hopelessness is essential to target among IPV survivors, and especially among formerly incarcerated African American IPV survivors (Fuentes, 2014). Optimally, such interventions will incorporate evidence-informed, gender sensitive, and culturally responsive strategies associated with cognitive-behavioral approaches and trauma-informed care (King, 2017; Lynch et al., 2012). The strategies should aim to reduce hopelessness through mitigating incarcerated and abused women’s negative self-concepts secondary to experiences with trauma and oppression and empower women through bolstering their self-compassion, self-efficacy, and capacity for self-advocacy. It also behooves behavioral health professionals to engage in advocacy and outreach to alter the contextual stressors common in the lives of the women they serve as psychological interventions do not reduce structural racism and the harms caused by the systemic criminalization of African American women who have survived abuse.

In closing, African American survivors of IPV often are negatively impacted by racist law enforcement and legal practices (Ritchie, 2017). As numerous reports and case studies show, the US legal system frequently fails IPV survivors of color (Richie & Eife, 2020). The very systems that are supposed to protect and support African American survivors instead have a legacy of criminalizing them (Richie & Eife, 2020; Ritchie, 2017). The destructive effects of criminal legal responses to communities of color have led to calls for providers and agencies to decrease their dependence on the criminal-legal system to protect girls and women from gender violence (Kim, 2018; Richie, 2012). Indeed, activists around the world recently have called for action to defund the police. Ultimately, responses that criminalize African American IPV survivors risk creating hopelessness, expanding gaps in services, and putting survivors at risk for repeatedly cycling through the legal system (Richie & Eife, 2020). IPV and harmful legal responses play a destructive role in the lives of African American women, leading to a sense of hopelessness and limited access to services. There is an urgent need to enhance methods that empower, rather than punish, African American survivors of IPV (Richie, 2012).
